# Skeleton-based cerebrovascular quantitative analysis

**DOI:** 10.1186/s12880-016-0170-8

**Published:** 2016-12-20

**Authors:** Xingce Wang, Enhui Liu, Zhongke Wu, Feifei Zhai, Yi-Cheng Zhu, Wuyang Shui, Mingquan Zhou

**Affiliations:** 1 College of Information Science and Technology, Beijing Normal University, Beijing, China; 2 Department of Neurology, Peking Union Medical College Hospital, Beijing, China

**Keywords:** Geometric factors, Circle of Willis, Quantitative analysis, B-spline curve, Skeleton, Stochastic analysis

## Abstract

**Background:**

Cerebrovascular disease is the most common cause of death worldwide, with millions of deaths annually. Interest is increasing toward understanding the geometric factors that influence cerebrovascular diseases, such as stroke. Cerebrovascular shape analyses are essential for the diagnosis and pathological identification of these conditions. The current study aimed to provide a stable and consistent methodology for quantitative Circle of Willis (CoW) analysis and to identify geometric changes in this structure.

**Method:**

An entire pipeline was designed with emphasis on automating each step. The stochastic segmentation was improved and volumetric data were obtained. The L1 medial axis method was applied to vessel volumetric data, which yielded a discrete skeleton dataset. A B-spline curve was used to fit the skeleton, and geometric values were proposed for a one-dimensional skeleton and radius. The calculations used to derive these values were illustrated in detail.

**Result:**

In one example(No. 47 in the open dataset) all values for different branches of CoW were calculated. The anterior communicating artery(ACo) was the shortest vessel, with a length of 2.6mm. The range of the curvature of all vessels was (0.3, 0.9) ± (0.1, 1.4). The range of the torsion was (−12.4,0.8) ± (0, 48.7). The mean radius value range was (3.1, 1.5) ± (0.1, 0.7) mm, and the mean angle value range was (2.2, 2.9) ± (0, 0.2) mm. In addition to the torsion variance values in a few vessels, the variance values of all vessel characteristics remained near 1. The distribution of the radii of symmetrical posterior cerebral artery(PCA) and angle values of the symmetrical posterior communicating arteries(PCo) demonstrated a certain correlation between the corresponding values of symmetrical vessels on the CoW.

**Conclusion:**

The data verified the stability of our methodology. Our method was appropriate for the analysis of large medical image datasets derived from the automated pipeline for populations. This method was applicable to other tubular organs, such as the large intestine and bile duct.

**Electronic supplementary material:**

The online version of this article (doi:10.1186/s12880-016-0170-8) contains supplementary material, which is available to authorized users.

## Background

**Fig. 1:**
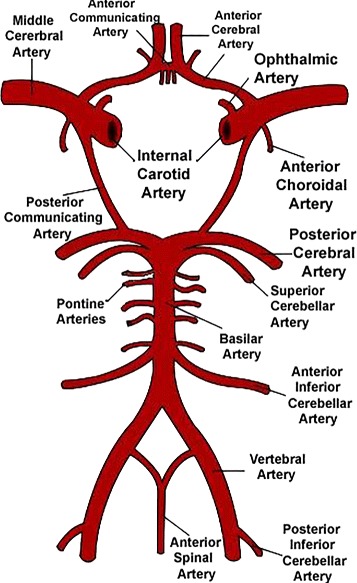
Schematic representation of the Circle of Willis. Image courtesy of wikipedia.org

Cerebrovascular disease is characterized by dysfunction of the blood vessels supplying the brain, resulting in stroke and subsequent disability or death. Subarachnoid hemorrhage, a life-threatening type of hemorrhagic stroke, is often caused by ruptured aneurysm or a weakened area in cerebral arteries. Since brain aneurysms often occur at the Circle of Willis(CoW)[1], the detection and quantitative analysis of CoW is essential for the prevention and treatment of aneurysm rupture and cerebral infarction. The construction of CoW is shown in Fig. 1 [2]. In the human brain, the CoW comprises the left and right anterior cerebral arteries(ACAs), the anterior communicating artery(ACo), the left and right middle cerebral arteries(MCAs), the left and right internal carotid arteries(ICAs), the left and right posterior communicating arteries(PCos), the left and right posterior cerebral arteries(PCAs), and the basilar artery(BA).

Rapid advances in medical technology have led to the development of diagnostic tools for cerebrovascular diseases, based on imaging [3]. Currently, three-dimensional(3D) image of the cerebral vasculature can be acquired routinely using three different imaging modalities: X-ray 3D rotational angiography(3DRA), computed tomography angiography(CTA), and magnetic resonance angiography(MRA). Time-of-flight(TOF) magnetic resonance angiography(MRA) via bright blood imaging is used to evaluate the intracranial vessels; since this modality does not require invasive procedures such as catheter angiography. However, the small but definite risk of embolism and complications, such as pseudoaneurysms, contrast-associated reactions and vascular dissections. TOF MRA was used in the present study. Reports suggested a certain amount of variation in both the geometry and topology of the CoW [4]. For example, some vessels may be duplicated, hypoplastic, or missing completely. A total of 83 variations in the CoW have been reported [5]. The vessels in the CoW are connected and twisted together, resulting in a complex topology. Furthermore, each vessel has a unique curvature and torsion and a variable radius, which together create a complex geometry.

Currently, most clinicians only use cerebrovascular volume data for visualization. Manual quantitative measurement of the vessel branches is a subjective and tedious task with a low level of reproducibility. Quantification and analysis of the cerebral artery geometry, particularly the arteries comprising the CoW, is of great interest. However, there remains a lack of knowledge about the geometric factors that can be used to differentiate between normal and pathologic vasculature or that are associated with endovascular treatment outcome. The geometric factors differentiating normal from pathologic vasculature or those associated with endovascular treatment outcome are unknown.

In addition, the challenges related to analysis of complex and variable vascular geometry of the CoW have led to the development of techniques and methodology for patient-specific geometric characterization of the CoW Quantitative measures would enable study and statistical analysis of the relationship between the local and global geometrical properties of the CoW, such as vessel curvature, tortuosity, length, radius, branching patterns and topology, as well as clinical events. Herein, we discuss the recent developments in CoW quantification.

Many studies have reported specific correlations between diseases and the geometry, as well as the topology of CoW. Gutierrez et al. reported a relationship between dolichoectasia(DE) and other diseases, and also provided DE diagnostic tools based on total cranial volume (TCV)-adjusted arterial diameter within two standard deviations(SD) [6, 7]. From a geometrical perspective, vessel dilatation, elongation and tortuosity might indicate various diseases(eg. ischemic, orhemorrhagic stroke or atherosclerosis). Vessel dilatation and elongation were characterized geometrically by the radius and length, respectively. Dilatation and elongation might also change the vessel volume and surface area. Research into DE conducted by Gutierrez reported arterial diameter measurements as well as the volume and length of basilar artery [7]. However, the diameters of individual vessels were calculated at the level of the largest cross-sectional area. Therefore, the diameter measurement was limited to a specific location. Furthermore, the study did not report specific methods for calculation of vessel volume or length. Other studies redefined DE by measuring the vessel length, local diameter and volume. For example, in some studies the diameters of individual vessels were calculated at either the level where the cross-sectional area was the largest or at another location of interest [8, 9]. In addition, Gutierrez et al. did not explicitly describe the method used to calculate diameters [10]. Kwon et al. and Maaly et al. determined the DE without calculating parameters such as length and diameter [11, 12]. Whereas, Lee et al. calculated volumes by measuring the cerebral blood flow [13]. Calculations based on length may be rough and less accurate. For tortuous vessels with angulations, length can be measured in segments by adding discrete distances [14]. However, the surface area of the vessel has only been calculated rarely in recent studies. Kim et al. reported a potential relationship between vascular tortuosity and intracranial artery atherosclerosis using the four parameters of the MCA: the diameter of the proximal MCA, the diameter of the distal the MCA, the length of the main trunk of the MCA and the minimal distance between the ends of the main trunk of the MCA [15]. Their measurements were based on slice data and did not use curvature or torsion to characterize vessel tortuosity. In another study, Jens et al. studied abnormal extracranial ICAs and used lateral cervical displacement to determine tortuosity [16]. Studies by Grisan et al. and Shelton et al. concerning vessel tortuosity measurements used retinal blood vessels to calculate the ratio of arc length to chord length and curvature [17, 18]. However, a few studies investigated tortuosity at other vascular sites or lesions, such as tumors, the cerebral microvasculature, coronary arteries and thoracic aorta [19–23]. Studies investigating the tortuosity of the CoW are very rare, particularly quantitative studies using 3D graphics rather than slice data. Blood vessels possess distinct tubular characteristics. The skeletal structure has a reduced dimensionality and shares same topology with the tubular shape. The skeleton comprises segments placed spatially along the medial region of the object and reflects the main geometrical features of that object. In a geometric representation, a tubular structure can be designed using two 1D measurements: the skeleton and vessel radii. Other quantities, such as vascular area and volume, can be calculated using these basic data. In our previous study, we used a new parameterized object geometric presentation proposed by Wu et al. for the vessels [24]. We reported how to quantify the CoW using the skeleton and vessel radii.

The main contributions of our paper are as follows:

1. We designed a pipeline for the 3D geometric quantification of the CoW, which yield a more accurate signature of the vessel based on cerebrovascular segmentation. Geometric vessel modeling and topology extraction were used to represent the vascular network of the whole brain. A semantic label was assigned to each branch using tree-based topology. The vessel skeleton and radius were calculated from the baseline data. Further stochastic analyses were performed using these data. The proposed pipeline generated more accurate and sophisticated data by comparing the traditional 2D projection image quantification and the vessel volume obtained from 3D quantification.
2. We used a B-spline curve for quantification of the vessel skeleton. The skeleton was calculated using the L1 normal method. Skeletons of the same vessel obtained using different sampling modes may have the similar shape but different discrete sets. The location of the discrete set caused fluctuations in length. In particular, the curvature and torsion varied, and the location of the discrete set introduced a large data error. Accordingly, a fitting step is necessary. We calculated the length, curvature and torsion of the skeleton and radius of the vessel, which together reflect the overall geometric parameters of the vessel. Other intrinsic parameters, such as the surface area and volume of the vessel, could be calculated using these basic data.

The rest of the manuscript is organized as follows. The materials and methods used in our study were discussed in Section methods and materials, including our method pipeline and experimental data. In Section geometric parameters of CoW, we describe the method used for vessel quantification. In Section results, the results of real TOF MRA and analytical experiments are presented. In Section discussion, we discuss the advantages and withdraws of the pipeline. Finally, we provide our conclusions and plans for future studies under Section Conclusions.

## Methods

### Materials

This study included data from 108 3D TOF MRI sub-study participants with available good-quality head MRA. The data used were obtained from the open source database http://public.kitware.com/Wiki/TubeTK/Data in the MHA format [25]. The 108 sets of data were obtained from healthy population, with similar size and resolution. The data were not labeled with client or disease information.

Data from subject number 47 were selected to illustrate the methodology used to calculate the different parameters and present the results with a size of 448×448×448 and resolution of 0.5×0.5×0.8*m* *m* ^3^. The No. 47 was a dataset pertaining to a 31-year-old female and her health status. The CoW from the patient was morphologically complete and unique applicable to each vessel of the CoW. The differences among the vessels are presented. The datasets supporting the conclusions of this article are included in the Additional files. We have partially analyzed the 108 sets. For all datasets, de-noising and regularity were the necessary pre-processing steps for subsequent study.

### Method pipeline

**Fig. 2:**
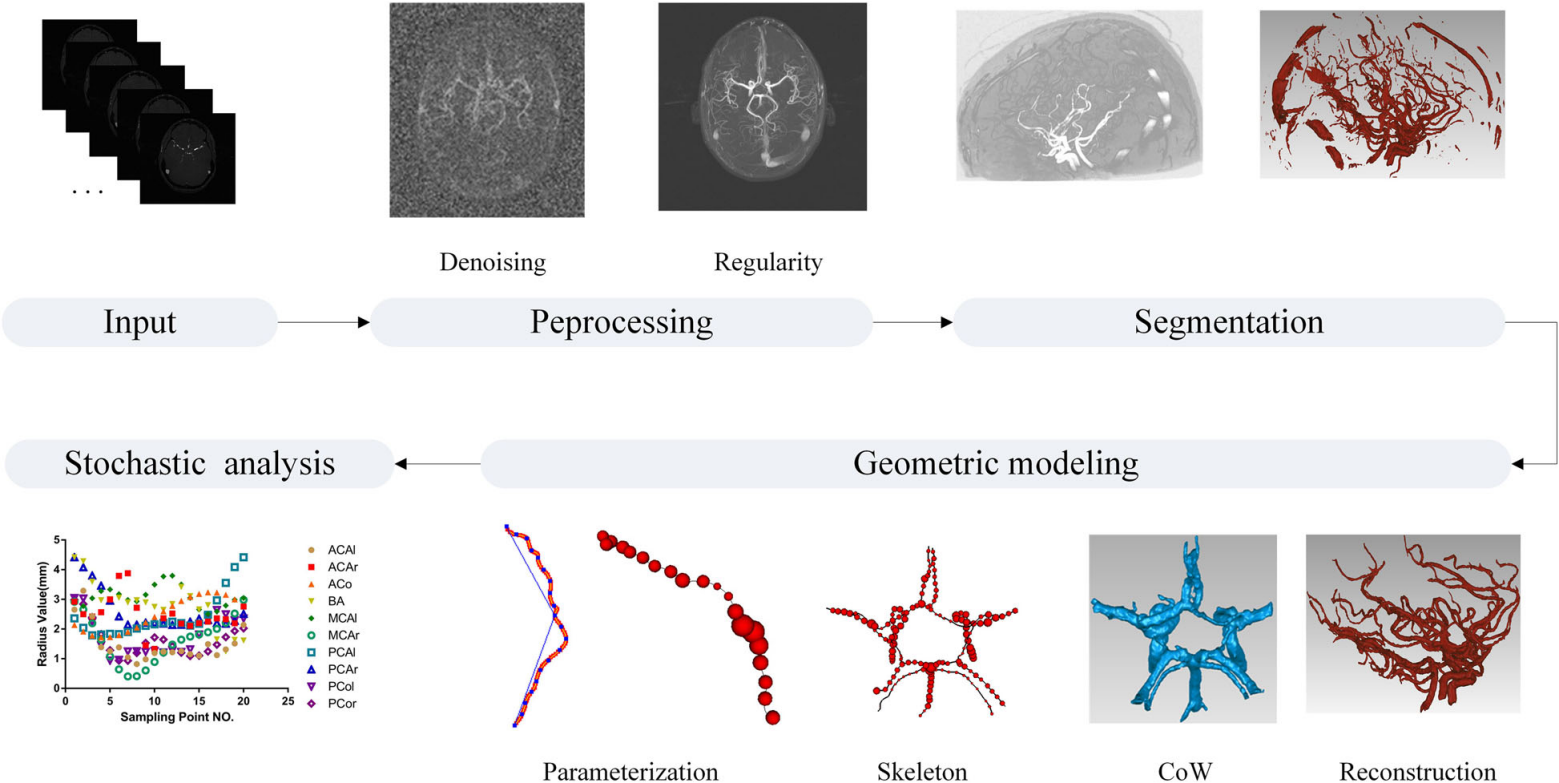
Pipeline for geometric CoW characterization

Vascular geometry plays an important motivational role in the design of processing pipelines for image-based, subject-specific geometry characterization. In general, angiographic data are readily available because of the widespread use of 3D imaging devices. However, there remains a lack of techniques that can be used to characterize cerebrovascular geometry and allow efficient data processing. A successful geometric characterization pipeline would consist of the following three elements (Fig. 2).

#### Vessel segmentation

The precise segmentation of the cerebrovascular vessels is essential for the quantitative analysis. However, the intensity contrast between a vessel and its surrounding tissues/organs may be very low on MRA images. This discontinuity exists in the vessels, and vascular regions occupy a small fraction of the whole image. Our previous study used an automatic statistical intensity-based approach to extract the 3D cerebrovascular structure with particle swarm intelligence algorithm [26], for accurate segmentation, particularly for small-sized blood vessels.

#### Geometric modelling extraction

Blood vascular data generated using medical imaging instruments(eg. CT, MRI) were initially represented as a volumetric model. Various geometry presentations are also applied according to the application; including polygonal mesh, non-uniform rational B-splines(NURBS) surface, implicit surfaces, and generalized cylinders. Since we are interested in the CoW, in the current study we determined the region of interest (ROI) by computing the L1-medial skeleton using an algorithm published by Huang et al. [27]. The L1-median was robust and the extracted curve skeleton was not sensitive to noise, outliers or missing data. Therefore, it faithfully revealed the one-dimensional structure of the brain vessel.

#### Characterization and statistical analysis

We described the geometry using a series of local or global features. The shape of a vessel skeleton is a rich source of geometric information. For example, the tortuosity metrics are of particular interest as a potential indicator of pathological development. In addition, the local curvature and torsion of the skeleton indicate the 3D bending of the vessel and the twisting of the curvature plane in space and are known to affect the underlying hemodynamic forces. In addition to the centerline shape, the local vessel diameter is related to the blood flow capacity, and the vessel cross-section area, mean thickness and volume are frequently used. These parameters facilitate the detection of hypoplasia and measurement of vascular thickness relative to other vessels. The above pipeline elements are illustrated with respect to geometric CoW as an example. All the elements were automated and were subjective and repeatable. The stability of the final statistical analysis of the large dataset was also determined.

## Geometric parameters of the CoW

The tree-based topology, was used to calculate different parameters of the vessel skeleton and radius. The skeleton of each branch is a discrete point set. An efficient fitting step is necessary for subsequent normal resampling of the skeleton. A B-spline curve was used to fit the initial discrete points and resample 20 points on the skeleton. The length, curvature, torsion and radius at these points were calculated as local vessel parameters. Global geometric parameters such as the vessel volume and surface area were then calculated using the basic data. In addition, the curvilinear integral of the curvature and torsion on each vessel skeleton were global geometric parameters for vessel labeling.

### B-spline fitting

The B-spline curve was used to fit the initial discrete point of one vessel skeleton, thus ensuring the continuity of the skeleton curve and verifying the accuracy of the calculation. The B-spline curve can be defined as follows:

1 $$ p\left(u \right) = {\sum\nolimits}_{i = 0}^{n} {{N_{i,k}}\left(u \right){p_{i}}}  $$

Here, *p*(*u*),*p*_*i*_ present 3D vectors:

2 $$ p\left(u \right) = \left({x\left(u \right),y\left(u \right),z\left(u \right)} \right)\qquad\qquad {p_{i}}=\left({x_{i}},{y_{i}},{z_{i}}\right)   $$

Where *p*_*i*_(*i*=0,1,⋯,*n*) are control points and *N*_*i*,*k*_(*i*=0,1,⋯,*n*) is the bias function. The profit-and-loss revision technique may improve the accuracy of approximation from the raw image data subject to cubic B-spline smoothing. The NURBS++ library was used to accomplish the fitting step.

Based on the B-spline function derivatives in the following description, B-spline higher derivative formulas were placed as follows:

3 $$ {{p}^{\left(r \right)}}\left(u \right) = {\sum\nolimits}_{i = 0}^{n - r} {{N_{i,k - r}}\left(u \right)} {p_{i}}^{\left(r \right)}  $$

4 $$ \text{with~}{p_{i}}^{\left(r \right)} = \left\{ \begin{array}{cc} {p_{i}} & r = 0\\ \frac{{k - r + 1}}{{{u_{i + k + 1}} - {u_{i + r}}}}\left({p_{i + 1}^{\left({r - 1} \right)} - p_{i}^{\left({r - 1} \right)}} \right) & r > 0 \end{array} \right.  $$

*p* ^(*r*)^(*u*) represents the *rth* derivatives of *p*(*u*). Therefore, ${p}\left (u \right) = {{p}^{\left (0 \right)}}\left (u \right) = {\sum \nolimits }_{i = 0}^{n} {{p_{i}}^{\left (0 \right)}{N_{i,k}}\left (u \right)}$ [28].

Then,

5 $$ \begin{aligned} &{{x}^{\left(r \right)}}\left(u \right) = {\sum\nolimits}_{i = 0}^{n - r} {{N_{i,k - r}}\left(u \right)} {x_{i}}^{\left(r \right)}\\ &\text{with}\quad {x_{i}}^{\left(r \right)} = \left\{ \begin{array}{cc} {x_{i}} & r = 0\\ \frac{{k - r + 1}}{{{u_{i + k + 1}} - {u_{i + r}}}}\left({x_{i + 1}^{\left({r - 1} \right)} - x_{i}^{\left({r - 1} \right)}} \right) & r > 0 \end{array} \right. \end{aligned}  $$

6 $$ \begin{aligned} &{{y}^{\left(r \right)}}\left(u \right) = {\sum\nolimits}_{i = 0}^{n - r} {{N_{i,k - r}}\left(u \right)} {y_{i}}^{\left(r \right)}\\ &\text{with}\quad {y_{i}}^{\left(r \right)} = \left\{ \begin{array}{cc} {y_{i}} & r = 0\\ \frac{{k - r + 1}}{{{u_{i + k + 1}} - {u_{i + r}}}}\left({y_{i + 1}^{\left({r - 1} \right)} - y_{i}^{\left({r - 1} \right)}} \right) & r > 0 \end{array} \right. \end{aligned}  $$

7 $$ \begin{aligned} &{{z}^{\left(r \right)}}\left(u \right) = {\sum\nolimits}_{i = 0}^{n - r} {{N_{i,k - r}}\left(u \right)} {z_{i}}^{\left(r \right)}\\ &\text{with}\quad {z_{i}}^{\left(r \right)} = \left\{ \begin{array}{cc} {z_{i}} & r = 0\\ \frac{{k - r + 1}}{{{u_{i + k + 1}} - {u_{i + r}}}}\left({z_{i + 1}^{\left({r - 1} \right)} - z_{i}^{\left({r - 1} \right)}} \right) & r > 0 \end{array} \right. \end{aligned}  $$

### Vessel Radius

**Fig. 3:**
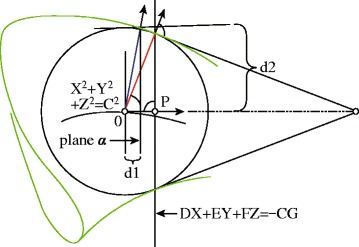
Computing the corresponding radius of vessel method

The vessel radius was calculated using the following method. The vessel region boundary can be regarded as the envelope surface of a one-parameter family of spheres (Fig. 3).

For any parameter *u*, the center curve and radius were calculated as follows:

8 $$ x\left(u \right) = {\sum\nolimits}_{i = 0}^{n} {{N_{i,{k}}}\left(u \right){x_{i}}}  $$

9 $$  y\left(u \right) = {\sum\nolimits}_{i = 0}^{n} {{N_{i,k}}\left(u \right){y_{i}}}  $$

10 $$ z\left(u \right) = {\sum\nolimits}_{i = 0}^{n} {{N_{i,k}}\left(u \right){z_{i}}}  $$

11 $$ r\left(u \right) = {\sum\nolimits}_{i = 0}^{n} {{N_{i,k}}\left(u \right){r_{i}}}  $$

12 $$ \begin{aligned} &{{r}^{\left(1 \right)}}\left(u \right) = {\sum\nolimits}_{i = 0}^{n - r} {{N_{i,k - r}}\left(u \right)} {r_{i}}^{\left(1 \right)}\\&{r_{i}}^{\left(1 \right)}=\frac{{k - r + 1}}{{{u_{i + k + 1}} - {u_{i + r}}}}\left({r_{i + 1}^{} - r_{i}^{}} \right) \end{aligned}  $$

Therefore,

13 $$ {\left({x - x\left(u \right)} \right)^{2}} + {\left({y - y\left(u \right)} \right)^{2}} + {\left({z - z\left(u \right)} \right)^{2}} - {\left({r\left(u \right)} \right)^{2}} = 0  $$

According to the envelope theorem, *F*(*x*,*y*,*z*,*u*)=0 and $ \frac {\partial F(x,y,z,u)}{\partial u}=0 $.

Therefore, (*x*−*x*(*u*))*x* ^(1)^(*u*)+(*y*−*y*(*u*))*y* ^(1)^(*u*)+*z*(−*z*(*u*))*z* ^(1)^(*u*)+*r*(*u*)*r* ^(1)^(*u*)=0

Let *X*=*x*−*x*(*u*), *Y*=*y*−*y*(*u*), *Z*=*z*−*z*(*u*), *C*=*r*(*u*), *D*=*x* ^(1)^(*u*), *E*=*y* ^(1)^(*u*), *F*=*z* ^(1)^(*u*), *G*=*r* ^(1)^(*u*).

Thus,

14 $$ {X^{2}} + {Y^{2}} + {Z^{2}} = {C^{2}}  $$

15 $$ DX + EY + FZ = - CG  $$

The solution of the above equation group is a circle that results from the intersection of a sphere defined by Eq. (14) and a plane defined by Eq. (15). Thus, the perpendicular vector from sphere centre O to the plane intersects at point P, and $\left |\overrightarrow {OP}\right | = - \frac {{CG}}{{\sqrt {{D^{2}} + {E^{2}} + {F^{2}}} }} $ The radius *R* of the vessel can be calculated conveniently, which corresponds to the skeleton data, as follows:

16 $$ R = \sqrt {{{{r^{2}}- \left|\overrightarrow{OP} \right|}^{2}}}  $$

### Length

Length is an important concept in the analysis of geometric shapes, and it is defined as the shortest distance between two points. Euclidean distance reflects only the relative spatial positions of two discrete points without any curve constraints. Arc length is the intrinsic character of a curve, and is not affected by a change in the coordinate system. Arc length is calculated using the following formula:

17 $$ L = \int\limits_{\Gamma} {ds}  $$

Where, *L* represents the arc length, *Γ* represents the arc and *d* *s* represents the micro-arc length on the arc. *L* can be obtained from the curve *Γ* integral.

With arc length parameterization, the curve presentation is unique and stable. Here, the length calculation is based on B-spline function integration and is equivalent to the integration curve arc. The integration curve arc formula is as follows:

18 $$ L = {\int_{0}^{1}} {\sqrt {{{\left({{x^{\left(1 \right)}}\left(u \right)} \right)}^{2}} + {{\left({{y^{\left(1 \right)}}\left(u \right)} \right)}^{2}} + {{\left({{z^{\left(1 \right)}}\left(u \right)} \right)}^{2}}} du}  $$

Where, *u* is the node vector, and its entire range is [0,1]. Thus, the integral interval of the curve is [0,1]. Accordingly, a more precise arc length can be derived.

### Curvature

Intuitively, the curvature is the amount by which a geometric object deviates from its position. Mathematically, the curvature is used to describe the camber degree at one point in a curve. The entire range of curvature is denoted by (0, +*∞*). A greater curvature indicates a greater degree of curve bending. For arc-length parametrization, the curvature at point p is defined as the rate of change of the curve angle bending with the arc length. Based on this definition, $ \kappa = \mathop {\lim }\limits _{\Delta s \to 0} \left | {\frac {{\Delta \theta }}{{\Delta s}}} \right | $, where *Δ* *θ* is the changed angle and *Δ* *s* is the changed arc length.

Thus, *κ*=∥*p* ^(2)^(*s*)∥.

The arc length parameter cannot be obtained in practice, But the arbitrary parameter of curvature can be obtained using with the Eq. (19) as follows:

19 $$  \kappa \left(u \right) = \frac{{\left\| {{p^{\left(1 \right)}}\left(u \right) \times {p^{\left(2 \right)}}\left(u \right)} \right\|}}{{{{\left\| {{p^{\left(1 \right)}}\left(u \right)} \right\|}^{3}}}}  $$

In Eq. (19), *p* ^(1)^(*u*)=(*x* ^(1)^(*u*),*y* ^(1)^(*u*),*z* ^(1)^(*u*)) and *p* ^(2)^(*u*)=(*x* ^(2)^(*u*),*y* ^(2)^(*u*),*z* ^(2)^(*u*)).

Thus:

20 $$ {\begin{aligned} &\kappa \left(u \right) =\\ &\frac{{\sqrt {{{\left({{z^{\left(2 \right)}} \cdot {y^{\left(1 \right)}} - {y^{\left(2 \right)}} \cdot {z^{\left(1 \right)}}} \right)}^{2}} + {{\left({{x^{\left(2 \right)}} \cdot {z^{\left(1 \right)}} - {z^{\left(2 \right)}} \cdot {x^{\left(1 \right)}}} \right)}^{2}} + {{\left({{y^{\left(2 \right)}} \cdot {x^{\left(1 \right)}} - {x^{\left(2 \right)}} \cdot {y^{\left(1 \right)}}} \right)}^{2}}} }}{{{{\left({{{\left({{x^{\left(1 \right)}}} \right)}^{2}} + {{\left({{y^{\left(1 \right)}}} \right)}^{2}} + {{\left({{z^{\left(1 \right)}}} \right)}^{2}}} \right)}^{\frac{3}{2}}}}} \end{aligned}}  $$

Here, *x* ^(1)^=*x* ^(1)^(*u*), *y* ^(1)^=*y* ^(1)^(*u*), *z* ^(1)^=*z* ^(1)^(*u*), *x* ^(2)^=*x* ^(2)^(*u*), *y* ^(2)^=*y* ^(2)^(*u*) and *z* ^(2)^=*z* ^(2)^(*u*).

### Torsion

The torsion of a curve measures how sharply it twists out of the plane of curvature. The entire range of torsion is (−*∞*,+*∞*). A greater absolute torsion value indicates a greater degree of curve warping. For arc-length parameterization, torsion at point *p* is defined as the rate of change of the osculating plane with the arc length. The following general parametric formula can then be obtained based on this definition and further derivation:

21 $$ \tau \left(u \right) = \frac{{\left({{p^{\left(1 \right)}}\left(u \right),{p^{\left(2 \right)}}\left(u \right),{p^{\left(3 \right)}}\left(u \right)} \right)}}{{{{\left\| {{p^{\left(1 \right)}}\left(u \right) \times {p^{\left(2 \right)}}\left(u \right)} \right\|}^{2}}}}  $$

Where *p*(*u*) represents the B-spline formula. Similarly, *p* ^(3)^(*u*)=(*x* ^(3)^(*u*),*y* ^(3)^(*u*),*z* ^(3)^(*u*)). *p* ^(1)^(*u*) and *p* ^(2)^(*u*) are as mentioned above.

We assume that *W*=(*p* ^(1)^(*u*),*p* ^(2)^(*u*),*p* ^(3)^(*u*)) and *V*=∥*p* ^(1)^(*u*)×*p* ^(2)^(*u*)∥.

Thus:

22 $$ \tau \left(u \right) = \frac{W}{{{V^{2}}}}  $$

23 $$ \begin{aligned} W &= {x^{\left(1 \right)}} \cdot y{}^{\left(2 \right)} \cdot {z^{\left(3 \right)}} + {z^{\left(1 \right)}} \cdot x{}^{\left(2 \right)} \cdot {y^{\left(3 \right)}} + {y^{\left(1 \right)}} \cdot z{}^{\left(2 \right)} \cdot {x^{\left(3 \right)}}\\ &- \left({z^{\left(1 \right)}} \cdot y{}^{\left(2 \right)} \cdot {x^{\left(3 \right)}} + {x^{\left(1 \right)}} \cdot z{}^{\left(2 \right)} \cdot {y^{\left(3 \right)}} + {y^{\left(1 \right)}} \cdot x{}^{\left(2 \right)} \cdot {z^{\left(3 \right)}}\right) \end{aligned}  $$

24 $$ {\begin{aligned} &V =\\ &\sqrt{{{\left({{y^{\left(1 \right)}} \cdot {z^{\left(2 \right)}} - {z^{\left(1 \right)}} \cdot {y^{\left(2 \right)}}} \right)}^{2}} + {{\left({{z^{\left(1 \right)}} \cdot {x^{\left(2 \right)}} - {x^{\left(1 \right)}} \cdot {z^{\left(2 \right)}}} \right)}^{2}} + {{\left({{x^{\left(1 \right)}} \cdot {y^{\left(2 \right)}} - {y^{\left(1 \right)}} \cdot {x^{\left(2 \right)}}} \right)}^{2}}} \end{aligned}}  $$

*W* and *V* are then included in the Eq. (22) to obtain the torsion of the point with a node vector *u*.

Here, *x* ^(3)^=*x* ^(3)^(*u*), *y* ^(3)^=*y* ^(3)^(*u*), *z* ^(3)^=*z* ^(3)^(*u*).

Overall, the curvature and the torsion of a space curve are analogous to the curvature of a plane curve. These parameters are coefficients in the system of differential equations for the Frenet frame derived from the Frenet-Serret formulaes. We believe that curvature and torsion can be used to describe the extent of vascular tortuosity.

### Included angle

**Fig. 4:**
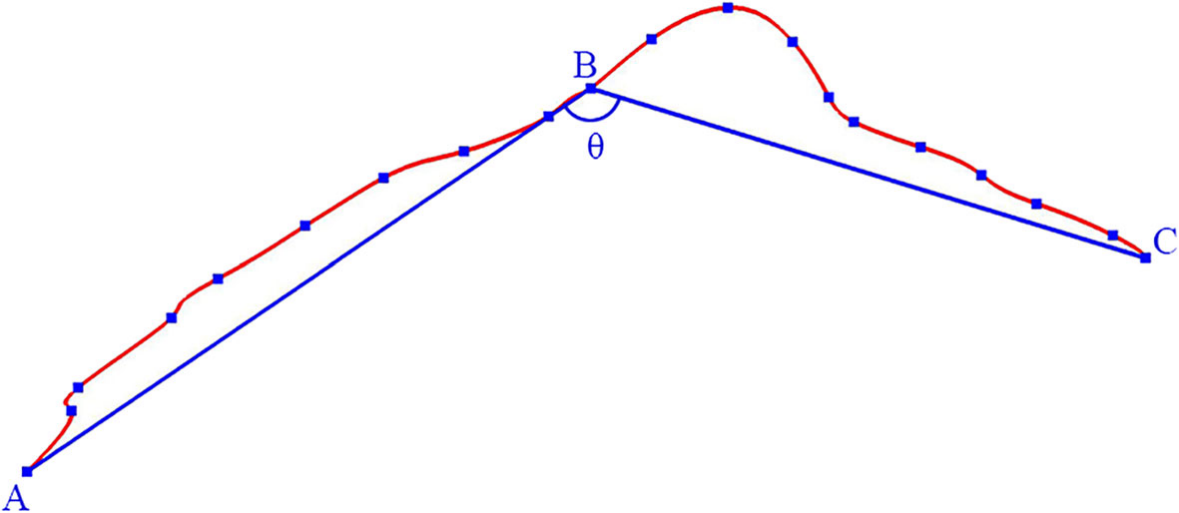
Angle comprising the vectors $\protect \overrightarrow {AB}$ and $\protect \overrightarrow {BC}$

As shown in Fig. 4, the red line is utilized to represent a blood vessel. Uniform sampling was performed along on the vessel. Point A represents the first point of the vessel, point C represents the last point of the vessel and point B represents one further sampling point in the vessel. For each vessel branch, the included angle was calculated between the vectors at the beginning point A, the sample point B and the endpoint C.

The scalar product of vector $\overrightarrow {AB}$ and vector $\overrightarrow {BC}$ was used to calculate the angle. This equation is shown in Eq. (25) and in Fig. 4.

25 $$ \overrightarrow{AB} \cdot \overrightarrow{BC} = \left| {\overrightarrow{AB}} \right|\left| {\overrightarrow{BC}} \right|\cos \theta  $$

This is a scalar quantity used to evaluate vessel tortuosity. In subsequent data analyses, it remained stable in the same vessel and varied in different vessels.

### Global parameters

In addition to the aforementioned parameters, the values of other parameters were determined, including vessel surface area and volume, skeleton arc-length curvature integration and torsion. The vessel surface area was calculated as the total area of all triangular facets in the segmentation data. Vessel volume was defined as the total volume of all small cubes represented by each coordinate point. Finally,the arc-length integration of the curvature and torsion were calculated from the integration-formulas of curvature and torsion on the arc. All these are global parameters of the semantic vessel, which can reflect the geometric parameters. All these parameters can be calculated using the primary data illustrated in the study. The calculation methodology for these parameters will be discuss in a subsequent publication.

## Results

The data from subject No. 47 were selected to illustrate the parameter calculation methodology and present the results. And also test and verify the effectiveness and enforceability of the method. All of the following results were derived from this sample.

### Data consistency

Under normal circumstances, physicians and radiologists prefer to evaluate the cerebrovascular volume using shape analysis. The topological structure is always analyzed by visual inspection, which yields rough quantitative data. The current study used the fitting method to reconstruct the cerebral vessels and calculate the geometric vessel parameters. It was first important to confirm that the results matched the raw data.

**Fig. 5:**
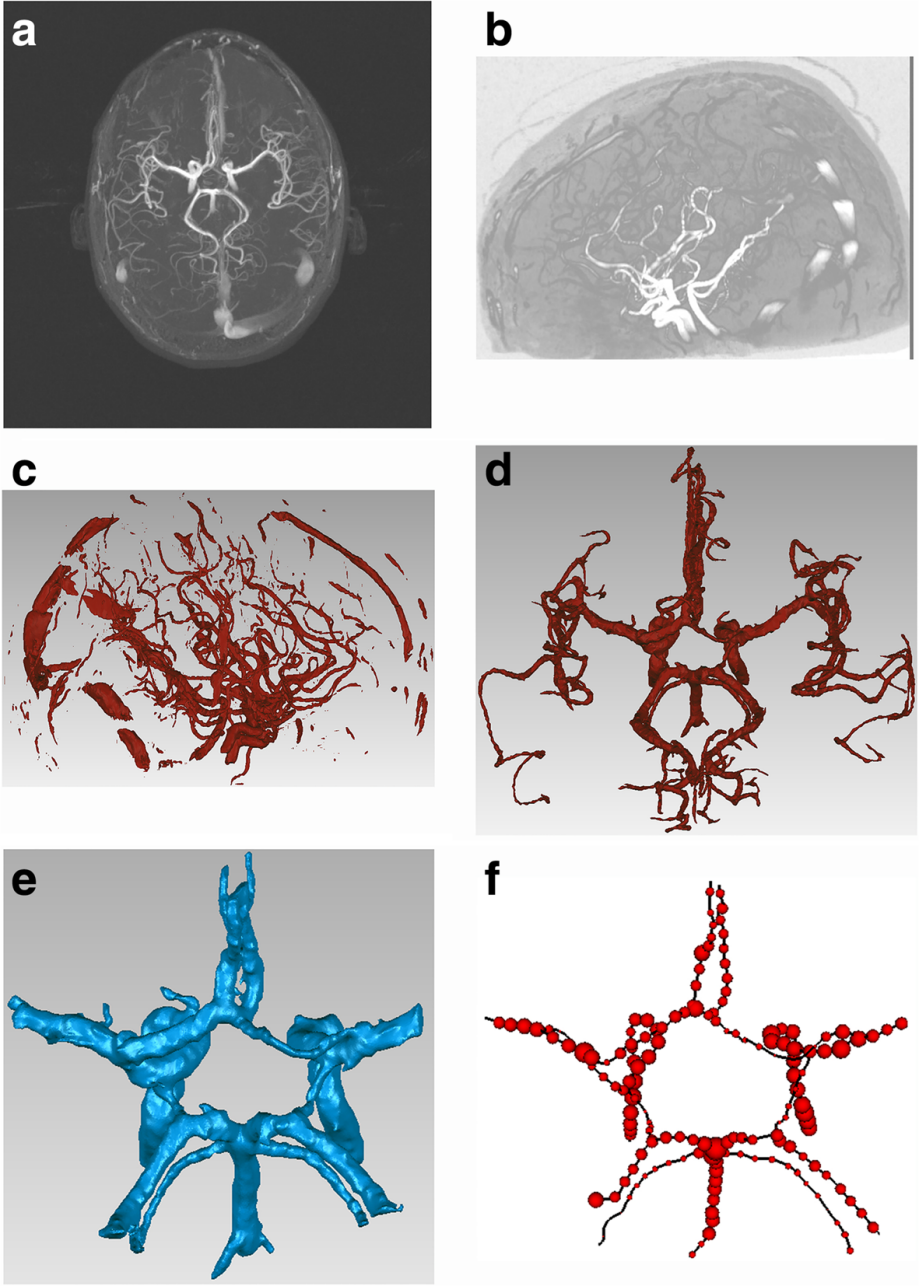
Pipeline results at different stages. **a** Maximum intensity project image. **b** Volume rendering result. **c** Cerebrovascular segmentation result. **d** Mesh reconstruction of the vessel. **e** CoW segmentation result with volume data. **f** B-spline curve of the COW skeleton and the corresponding radii of the sample data

In Fig. 5, it shows pipeline results at different stages. The maximum intensity project image is shown in Fig. 5 a; the CoW is very distinct and is used for diagnosis. The volume rendering is shown in Fig. 5 b, with the vessel shown in high intensity. The cerebrovascular segmentation results are shown in Fig. 5 c, which suggest a favorable shape and structural connectivity. The tissue of CoW is unbroken and clear. VTK was used to realize a mesh reconstruction of the vessel (Fig. 5 d).

The CoW was delineated from the cerebrovascular segmentation results, based on a rough guideline. The segmented tissue surrounding the CoW was rendered as smooth and precise as possible when it was cut. Messy small blood vessels were resected, as were insignificant vessels with severe bending. The length of the two branch vessels that intersected both ends of the ACo was 20 mm. The length of the two branch vessels(parts of the MCAs) that intersected with the nodes of ACAs and ICAs was 6.5 mm. The length of the two branch vessels that intersected with the nodes of the PCos and PCAs was 15 mm and the two branch vessels that intersected with the nodes of the BA and PCAs was nearly 22 mm long. The remaining parts of the ICAs and the BA maintained their original state. The ICAs described the ICA section in the CoW, corresponding C1 of the ICA.

The results of CoW segmentation using the mesh data reconstruction results are shown in Fig. 5 e. The B-spline curve of the CoW skeleton and corresponding radii of the sample data are shown in Fig. 5 f. These fitting results could then be used to determine the quantitative 1D data values (skeleton and radii) for further analysis. Based on these images, revealed that the geometric consistency of the CoW was visibly preserved in the pipeline.

**Fig. 6:**
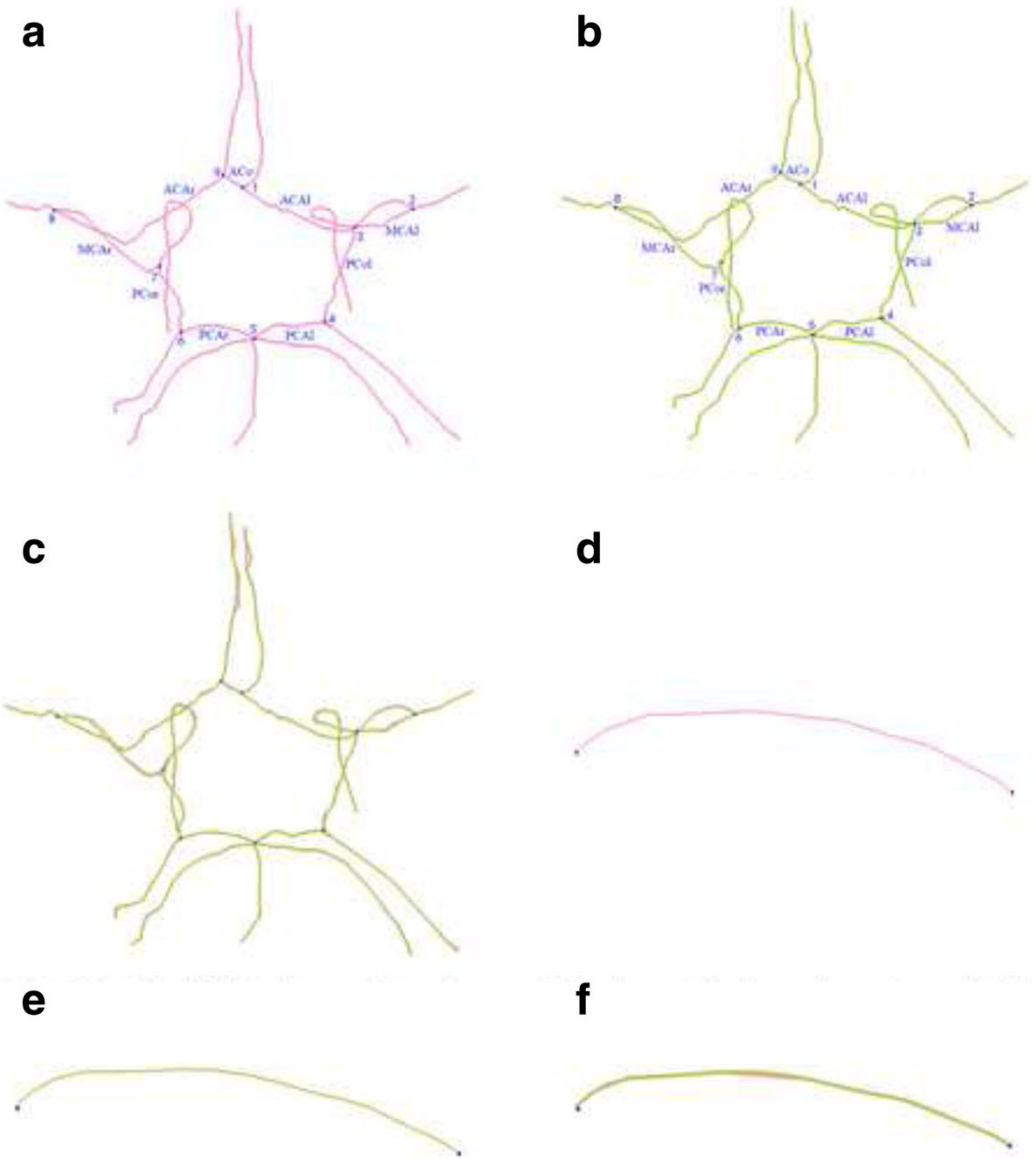
Consistency between the initial skeleton data and the B-spline skeleton data. **a** Initial skeletal topology. **b** B-spline skeleton topology. **c** Matching of the initial skeleton topology and B-spline skeleton topology. **d** Line image of the discrete data points on the initial skeleton of PCAr. **e** Line image of 20 sampling points on the B-spline skeleton of PCAr. **f** Matching of discrete data points from the initial skeleton and 20 sampling points on the B-spline skeleton of PCAr

Based on cerebrovascular segmentation and skeleton, tracking bifurcation points were used to create a graph structure. To identify the vessel branches, a topology was constructed using the tracking method, and vessel bifurcations were subsequently obtained, which ultimately define the vessel network. The Support Vector Machine (SVM) was used to classify the parent and child nodes of the bifurcations. A breadth first search(BFS) was used to identify these nodes accordingly and ultimately renderred the resulting structure. Because the CoW is the only one one-genus organ of the vessel, the CoW component can be easily identified in the vessel network. After registration with the CoW atlas [29], the semantic labels for different branches in CoW red could be obtained and the B-spline curve was used to fit these branches. The consistency between the initial skeleton and the B-spline skeleton data in shown in Fig. 6. The initial skeleton topology data of No. 47 is shown in Fig. 6 a together with the transverse section. The branch point is indicated by the blue point. The skeleton B-spline fitting result is shown in Fig. 6 b. Each branch was labeled with an anatomic designation, as shown in Fig. 6 a, b. The aligning method was then used to combined the initial skeleton curve and fitting curve with the B-Spline curve in Fig. 6 c.

The initial skeleton topology data were determined for No. 47, which is shown as a transverse section in Fig. 6 a. The skeleton B-spline fitting result is displayed in Fig. 6 b. Figure 6 c revealed see that the shapes of these two curves were almost identical in shape. This reflects both the geometric parameters of the CoW in No. 47 and the skeleton B-spline fitting result, which almost entirely covered the initial skeleton data. Furthermore, eight sampling points were collected at uniform specific lengths from the initial skeleton data of each individual CoW vessel. The data were used to calculate the minimum distances, or errors, between the sampling points and the B-spline fitting result on all individual CoW vessels as a measure of the effectiveness of the B-spline fitting. Figure 6 d depicts a line drawing of the initial discrete PCAr point data from the skeleton. Figure 6 e shows a line drawing of 20 sampling points on the B-spline skeleton of the PCAr. Finally, Fig. 6 f shows matching data from the initial skeleton discrete points and 20 sampling points on the B-spline skeleton of the PCAr. The two figures also partially illustrate the consistency between the initial skeleton data and the B-spline fitted skeleton for this single PCAr.

Table 1 shows the sums, mean values and variance in the minimum distances between the B-spline skeleton and eight sampling points in the initial skeleton data of every CoW vessel from the NO.47. All numeric values in Tables 1, 2, 3, 4, 5 and 6 were cited up to two decimal places. Table 1 reveals that the sums of the errors of each vessel in the CoW were located within the range of (0.1, 1.3) mm, the mean errors were in the range (0, 0.2) mm, and the variation of the error was within (0, 0.1) mm. The errors were already extremely small. Meanwhile, the sum, the mean and variance of the PCAr were minimal, whereas those of the PCol were maximal. In other words, the B-spline result of the PCAr and the PCol were the best and the worst fitting result among all the CoW branch vessels respectively. These errors could be caused by severe local tortuosity. However, the tendency and shape of the initial skeleton and the B-spline fitting skeleton were consistent, and these errors were insignificant and had no effect on the calculated parameters.

**Table 1:** Difference between the B-spline skeleton and initial skeleton of each vessel in the CoW

	*Error (mm)*									
	ACo	ACAl	ACAr	ICAl	ICAr	PCol	PCor	PCAl	PCAr	BA
Sum	1.14	0.63	0.91	0.88	1.28	1.31	0.28	0.66	0.06	0.49
Mean	0.14	0.08	0.11	0.11	0.16	0.16	0.04	0.08	0.01	0.06
Variance	0.02	0.01	0.01	0.01	0.01	0.04	0	0.01	0	0

**Table 2:** The length of each individual vessel from the No. 47 CoW dataset

	ACo	ACAl	ACAr	ICAl	ICAr	PCol	PCor	PCAl	PCAr	BA
Length (mm)	2.62	23.47	25.11	7.05	17.21	17.18	12.82	12.06	10.31	24.10

**Table 3:** Mean curvature and variance for all sampling points of each individual vessel from the No. 47 CoW dataset

	*Curvature*									
	ACo	ACAl	ACAr	ICAl	ICAr	PCol	PCor	PCAl	PCAr	BA
Mean	0.45	0.68	0.88	0.81	0.27	0.68	0.42	0.68	0.43	0.30
Variance	0.05	1.26	1.17	0.72	0.05	0.52	0.13	1.40	0.32	0.11

**Table 4:** Mean torsion and variance for all sampling points of each individual vessel from the No. 47 CoW dataset

	*Torsion*									
	ACo	ACAl	ACAr	ICAl	ICAr	PCol	PCor	PCAl	PCAr	BA
Mean	−1.20	−0.08	−1.41	−0.13	−0.02	0.82	−0.13	−2.36	−0.30	0.41
Variance	11.02	0.60	50.23	6.75	0.04	8.22	1.67	48.72	4.59	31.21

**Table 5:** Mean radii and variance for all sampling points of each individual vessel from the No. 47 CoW dataset

	*Radius(mm)*									
	ACo	ACAl	ACAr	ICAl	ICAr	PCol	PCor	PCAl	PCAr	BA
Mean	2.48	1.50	2.49	3.08	1.65	1.79	1.63	2.42	2.62	2.82
Variance	0.33	0.40	0.38	0.14	0.65	0.50	0.34	0.57	0.52	0.67

**Table 6:** Mean included angles and variance for all sampling points of every individual vessel from the No. 47 CoW data set

	*Angle*									
	ACo	ACAl	ACAr	ICAl	ICAr	PCol	PCor	PCAl	PCAr	BA
Mean	2.89	2.15	2.27	2.82	2.27	2.25	2.44	2.60	2.24	2.53
Variance	0.02	0.06	0.01	0.01	0.15	0.06	0.11	0.04	0.17	0.04

### Parameter calculation

**Fig. 7:**
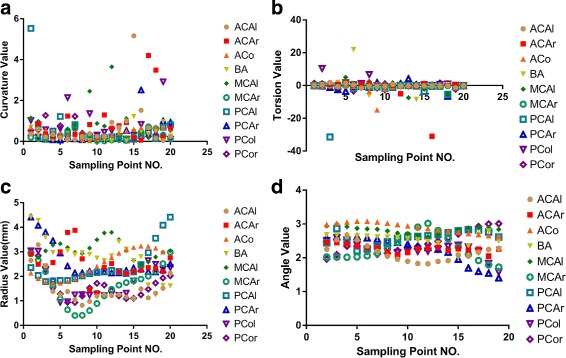
Parameters calculated using the current method in the sample No. 47 CoW dataset base on the following TOF MRA data: size of 448 ×448×128 and a resolution of 0.5 ×0.5× 0.8 *m* *m* ^3^. **a** Distribution of the sampling point curvature values on the individual vessels. **b** Distribution of the sampling point torsion values on the individual vessels. **c** Distribution of the sampling point radius values on the individual vessels. **d** Distribution of the sampling point angle values on the individual vessels

For each semantic branch of the fitting results, 20 points with the same arc length were sampled uniformly. The sample points were numbered 1–20 in a clockwise pattern and the curvature, torsion, radii and included angle were calculated. The parameters are shown in Fig. 7.

Because of the uniform sampling of individual vessels based on length, the distribution of the length values of each individual vessel exhibited a linear trend. Therefore, the length value distributions of the sampling points on the individual vessels were not presented. As shown in the above figures, the general parameter values could be analyzed from the sampling points of each individual vessel in the CoW, including the approximate parameter ranges, the primary distribution trends and abnormal analyses. As shown in Fig. 7 a, most curvature values were distributed between 0 and 1 with a particular bias toward 0, suggesting that the curvature radius at the sampling point was relatively large.

This revealed that the shape of the overall blood vessels were smooth and that they did not exhibit severe bending. Figure 7 b shows that very few sampling points on different vessels appeared to be outliers and that most torsion values on the CoW vessels were distributed near 0. If the torsion value of each point on the plane curve was 0, the torsion values were close to 0 and the degree of spatial distortion was lower. From the perspective of torsion, the CoW vessels do not were smooth without any distortion. In Fig. 7 c most radii were located within the interval of (1.0, 3.0) mm, with fluctuation in corresponding sampling points. In Fig. 7 d most of the angle values were located within the interval of (2.0, 3.0) and were close to *π*. This result suggested that the vessels were smooth, and that there were no overtly significant fluctuations in the overall pattern of the CoW blood vessels. The distribution of the angle values resembled that of the curvature values. A larger angle value is associated with a larger curvature radius and smaller curvature. The distribution pattern suggested that most curvature values were near 0, with angle values near *π*.

Table 2 shows the length of each individual vessel in the CoW. The values declined within the range of (2.6, 24.1) mm. Table 3 reveals that the ACo was the shortest vessel in the CoW. The difference in length between ICAl and ICAr might be directly attributed to differences in vascular morphological. Tables 4, 5 and 6 present the mean values and variation for every parameter of each individual vessel in the CoW, which are based on all sampling points. In Table 3, the mean curvature of each vessel on CoW fell within (0.3, 0.9). The mean ICAr and BA curvature values were smaller than those of other vessels, whereas the mean ACAr curvature was the highest. This suggests that ICAr and BA are smoother than the other vessels, whereas the ACAr contains more bends. Combined with the variance, a smaller difference indicates that the curvature value at each sampling point is close to the mean curvature. The variances in the ICAr and ACo were lower than those of other vessels; the curvature of each ICAr sampling point was close to the mean as 0.3, whereas the curvature of each ACo sampling point was close to the mean as 0.5. The PCAl variance value represented the maximum value; therefore, the curvature of a few PCAl sampling points represent significant deviations from the mean, resulting in distinct fluctuations. Generally, the variation in each vessel was small, and the curvatures of each point on CoW were centralized. Table 4 shows the mean torsion value and the variation in each vessel in the CoW. The mean torsion values fell within the interval (-2.4, 0.8), and none of the vessels in the CoW exhibited severe spatial rotation. Unlike the curvature, the variance of specific vessels were relatively large, and there was a substantial difference between the torsion values of these vessels. However, the torsion variance of specific vessels(eg. the ACAl,the ICAr,and the PCor) remained near 1, indicating relatively uniform space rotation rates for these vessels. Table 5 shows that the maximum and mean radii were 3.1 mm and 1.5 mm, respectively. All vessels showed variance values within the interval (0.1, 0.7) mm, with relatively stable CoW radii. In Table 6, the mean angle values fell within the interval (2.2, 2.9), and the angle variance fell within the interval (0, 0.2). This further suggests that all the CoW vessels were relatively smooth.

**Fig. 8:**
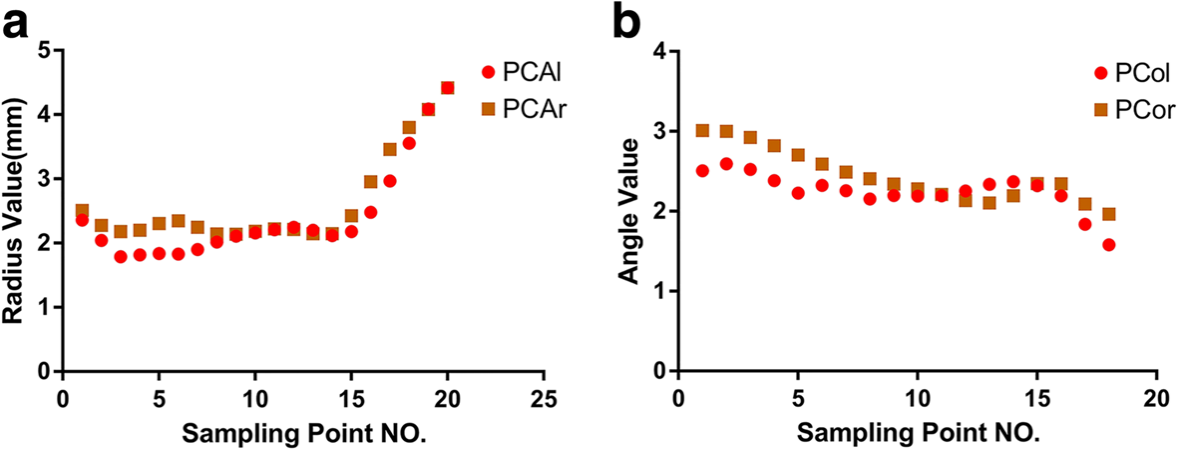
Parameters distributions for the symmetrical vessels in the CoW. **a** Distribution of sampling point radius values for the PCAl and PCAr. **b** Distribution of sampling point angle values for the PCol and PCor

Since the CoW is an approximately symmetrical structure, it is possible that the corresponding parameters of the symmetrical vessels on the CoW, such as the PCAs(PCAl and PCAr), PCos(PCol and PCor) and ACAs(ACAl and ACAr), are specifically correlated with each other, which are shown in Fig. 8. Therefore, the radii of both PCAs and the angles of both PCos were measured as an example to illustrate the relationship between symmetrical vessels on the CoW. As shown in Fig. 8 a, the radii of symmetrical sampling points on the PCAs were very similar, and the distributions of the radii of the sampling points were nearly identical. However, a few sampling points’ radii increased at the end. Similarly, Fig. 8 b shows that the angles of symmetrical sampling points on the PCos were very similar, and that the distributions of the angle of the sampling points were almost identical. The angle became smaller for the later sampling points, which suggests greater curvature in the tails of the PCos. The resolution of the MRA image was determined up to one decimal places.

However, for the convenience, all the values presented in the paper were indicated up to two decimal places. All the other data from subject number 47 are shown in Additional files 1, 2, 3, 4, 5 and 6. For the detail visualization, please refer them.

## Discussion

The quantification and analysis of cerebral artery geometry, particularly the arteries that constitute the CoW, is of great interest [30].

Pathological changes in the cerebral arteries mainly appear in three patterns: 1) lumen stenosis of the cerebral arteries due to atherosclerosis [31], 2) DE and tortuosity caused by injury to the intima or elastic tissue in the tunica media vasorum [32], and 3) vascular stiffness due to loss of elastic fibers in the vascular wall [33].

Currently, neurologists and radiologists not only focus on the cerebral artery stenosis, but also are interested in delineating the geometric characteristics of the arteries, such as elongation, dilation and tortuosity. Previous studies [34–37] correlated dilatative arteriopathy with cerebral vascular disease. Identifying these factors was mainly based on reading manual images and vessel diameter measurements, with different scale. However, the consistency, accuracy, and efficiency of this method cannot be verified. Stability, accuracy, and efficiency all need to be analyzed in subsequent studies. In the current study, we developed a skeleton-based automated segmentation algorithm using a TOF MRA dataset, which provided different geometric parameters to evaluate the vascular structure including radius, length, curvature, torsion, and the included angle. This method could be applied to other cerebral arteries, such as the basilar artery or internal carotid artery, to investigate the association between artery geometric structure of arteries and cerebral vascular disease.

VMTK can be used to facilitates the determination of the centerline of the corresponding vessel, but manually selection the source and target points is tedious and time-consuming. The current method is fully automated and eliminates human error. In addition, the radius obtained using the current method and manual measurements on 2D slice data by a physician were compared. The results suggested that the automated calculations are realistic and valid.

Radiological data and the current methodology can be combined to analyze vascular features numerically and present significant parameters in the vascular network based on an explicit numerical index. This quantitative vascular analysis enables vascular anomalies to be identified and the risk of vascular disease to be predicted without relying on the subjective judgement of physicians [38, 39]. Early vascular quantitative analysis focused mainly on retinal vessel network [40]. The computerized analysis of retinal vessel width and tortuosity in premature infants study attempted to determine the risk of retinopathy of prematurity (ROP) using digital retinal images of infants (n = 16). Kandasamy et al. [41] extended this work to include retinal microvasculature measurements in full-term newborn infants (20 infants; 9 female and 11 male). Bullitt et al. [25] first used high-resolution MRA data to evaluate abnormal tortuosity, and offered a novel approach for the non-invasive diagnosis of malignant tumors by comparing the regional vasculature in 34 healthy subjects with the tumor-associated vasculature in 30 brain tumors before surgical resection. In another study, Hu et al. [42] reconstructed the angioarchitecture in normal and injured subjects using micro-CT images of silicone rubber microsphere-perfused specimens, based on the hypothesis that the spinal cord would undergo reorganization and post-injury modification of the vascular networks after acute spinal cord injury. This method contributed to the elucidation of the mechanisms underlying compensatory vascular reconstitution in the traumatized spinal cord. In China, Xiang et al. [43] used synchrotron radiation to calculate the microvessel density and fractal dimensions of tumors. These authors analyzed neovascular tumor morphology at different stages.

The numerical index of a vessel directly influences the result of quantitative analysis. However, many diseases can affect the distribution, structure and morphology of a vessel before clinical manifestation. A numerical index can be used to express the geometric and topological features of the vessel network data. An abnormal vessel network may exhibit great differences in these numerical indices, compared with a normal vessel network. Therefore, these data can be used to evaluate the pathological changes to diseases diagnose and facilitate the relevant disease and geometric risk factors.

In the context of previous work, the current study presents the first quantitative analysis of vessels using computer graphics. Firstly, the pipeline for quantitative brain vessel analysis was confirmed. Many unique technologies are used in the pipeline. For example, stochastic method was applied to cerebrovascular segmentation, B-spline curve was used to present vessel skeletons and tree data structures revealed the topology of the brain vessels. Next, a numerical index style was designed for 1D calculations(eg. skeletons and radii). Each vessel is a tubular shape, and richly detailed information is contained within the skeleton and radii. To our knowledge, no previous studies have investigated tortuosity in the CoW, although such work is normal in the context of the aorta. Most studies calculated the ratio of the arc length and chord length, which is an inaccurate method. To date,no studies have incorporated skeletal curve torsion or the angle spanning the beginning, sample and end points.

Although the DE is a normal topic in the field of vessel research, measuring vessels length manually is the standard method; and automated geometric measurements are used less frequently. Finally, all the numerical indices were calculated for the sample points in the CoW in the study sample. The calculation of this single dataset lasted less than 10 min as all operations. The automated calculations for the skeleton and topology accounted for most of this time. The current algorithm and pipeline indicate the fundamental efficiency of large population-based calculation and analysis.

In the current study, we described a quantitative method for the arteries, using a validated dataset of the complete CoW. Future studies will investigate the relevance of the geometric numerical indices of different branches in the CoW. Future studies will focus on exclusive geometric findings related to risk factors such as aging, hypertension, diabetes, smoking and pregnancy, which are systemic conditions that influence the entire vasculature. Studies will also aim to elucidate the most likely geometric difference in the CoW related to stroke or aneurysm in a Chinese population. Perhaps future research will allow additional numerical vascular indices to be identified in clinical application. Currently, the lack of appropriate techniques is a limitation in investigating these data.

The current method can be used to determine the relationship between vascular geometry and predict the topological structure of the incomplete CoW associated with anatomical variants and embryological remnants. Currently, this method is great significance research interest and hasn’t yet reached clinical application. However, combining this method with an appropriate clinical imaging technique may be an option. A few studies in our laboratory explored the incomplete CoW with machine learning, which will be discussed in the future.

In spite of the promise of this new method, it is also associated with some problems and challenges. For example, the resolution and the image sequences may vary significantly in different datasets. In addition, the estimation parameters and fitting error for the segmentation method and B-spline fitting method may be altered based on a new dataset. The automated topology recognition needs to be further optimized. Nevertheless, we believe that the robust nature of the L1 method means that good results can be obtained on a large dataset.

It should be noted that the current study only addresses the geometric factors in one example data set. Consistency with physician judgment should be verified in a double blind study. These results should also be extended to a larger experimental analysis. Accordingly, future studies will investigate open source data and Chinese large-population data.

## Conclusions

The current study focused on the development and validation of a method for the image-based geometric characterization of the CoW. It covers the entire processing pipeline from a medical image to the extraction of geometric descriptors, thus facilitating each branch to be calculated.

First, a full flow chart of the geometric characterization of the vessel was designed. Next, the B-spline curve was used to fit the discrete skeleton and define four geometric characters. The CoW was characterized using different approaches to integrate values: one-order derivative values and two-order derivative values. The methodology for determining these parameters is presented clearly using B-spline curve fitting skeletons. All the parameters of the CoW were calculated in one example data set, and a simple analysis was provided to verify the effect of the aforementioned pipeline. Future studied will extend this work to clinical and population-based research, and provide a quantitative analysis of disease based on the methodology developed.

## Additional files

Additional file 1 Normal047-MRA.mha. This file is the original file of NO.47 data stored by.mha format which is compressed by DICOM series data. It is the initial data and the foundation of the follow-up data (e.g. the segmentation data, the skeleton data). (MHA 24064 kb)

Additional file 2 Normal047-segmentation.obj. This file is the segmentation result of the NO.47 data based on the Additional file 1: Normal047-MRA.mha. (OBJ 2846.72 kb)

Additional file 3 Normal047-mesh.obj. This file is the mesh data of the segmentation result and only contains the CoW. (OBJ 25702.1kb)

Additional file 4 Normal047-skeleton.obj. This file is the skeleton data of the NO.47 data based on the Additional file 2: Normal047-segmentation.obj and cleansed of noise points except the CoW. (OBJ 13.4 kb)

Additional file 5 Normal047-B-splineControlPoints.docx. This file contains ten tables (Table S1–S10). Every table records the control points of the corresponding vessel on the CoW after B-spline fitting. (DOCX 24.2 kb)

Additional file 6 Normal047-Characters.docx. This file contains four tables (Table S11–S14). Table S11 records the curvature values of twenty sampling points of each vessel on the CoW. Table S12 records the torsion values of twenty sampling points of each vessel on the CoW. Table S13 records the radius values of twenty sampling points of each vessel on the CoW. Table S14 records the angle values of eighteen sampling points of each vessel on the CoW. The data of these four tables correspond to the Fig. 7 in the paper. And the data of the Fig. 8 are contained in all these four tables. (DOCX 26.9 kb)

## Abbreviations

3D: Three-dimensional
3DRA: 3D rotational angiography
ACAl: Left anterior cerebral artery
ACAr: Right anterior cerebral artery
ACAs: Anterior cerebral arteries
ACo: Anterior communicating artery
BA: Basilar artery
BFS: Breadth first search
CoW: Circle of willis
CT: Computed tomography
CTA: Computed tomography angiography
DE: Dolichoectasia
ICA: Internal carotid artery
ICAs: Internal carotid arteries
ICATB: Internal carotid artery to the terminal bifurcation
MCAl: Left middle cerebral artery
MCAr: Right middle cerebral artery
MCAs: Middle cerebral arteries
MRA: Magnetic resonance angiography
MRI: Magnetic resonance imaging
NURBS: Non-uniform rational B-splines
PCAl: Left posterior cerebral artery
PCAr: Right posterior cerebral artery
PCAs: Posterior cerebral arties
PCol: Left posterior communicating artery
PCor: Right posterior communicating artery
PCos: Posterior communicating arteries
ROI: Region of interest
ROP: Retinopathy of prematurity
SD: Standard deviations
VTK: The Visualization Toolkit
VMTK: The Vascular Modeling Toolkit
TCV: Total cranial volume
TOF: Time-of-flight
