# Supplementary material for: Skeleton-based cerebrovascular quantitative analysis
Source: BMC Med Imaging. 2016 Dec 20;16:68. doi: 10.1186/s12880-016-0170-8 (PMC5168872; doi:10.1186/s12880-016-0170-8)
Supplement: Additional file 5 — Normal047-B-splineControlPoints.docx. This file contains ten tables (Table S1–S10). Every table records the control points of the corresponding vessel on the CoW after B-spline fitting. (DOCX 24.2 kb) [file 12880_2016_170_MOESM5_ESM.docx]

Table S1.The control points of B-spline fitting ACAl of NO.47 data

| ***ACAl*** | | | |
| --- | --- | --- | --- |
| **NO.** | **x** | **y** | **z** |
| 1 | 101.677 | 90.8831 | 21.5646 |
| 2 | 102.171 | 90.3623 | 22.7787 |
| 3 | 104.79 | 89.9842 | 22.8235 |
| 4 | 106.073 | 91.7537 | 23.6091 |
| 5 | 107.16 | 92.6929 | 24.4835 |
| 6 | 108.275 | 94.1127 | 25.088 |
| 7 | 110.201 | 94.1693 | 25.8872 |
| 8 | 111.888 | 93.2292 | 27.4292 |
| 9 | 112.862 | 92.755 | 26.0023 |
| 10 | 114.05 | 91.8467 | 26.6537 |
| 11 | 114.315 | 92.2307 | 23.9337 |
| 12 | 114.918 | 91.1184 | 25.323 |
| 13 | 115.742 | 91.1903 | 23.6007 |
| 14 | 116.588 | 90.031 | 23.015 |
| 15 | 117.67 | 89.3559 | 22.2837 |
| 16 | 118.143 | 88.7683 | 21.8837 |

Table S2.The control points of B-spline fitting ACAr of NO.47 data

| ***ACAr*** | | | |
| --- | --- | --- | --- |
| **NO.** | **x** | **y** | **z** |
| 1 | 137.219 | 90.5544 | 19.8926 |
| 2 | 136.699 | 90.7186 | 20.8515 |
| 3 | 135.906 | 91.2584 | 20.4317 |
| 4 | 134.622 | 91.7625 | 19.6181 |
| 5 | 133.867 | 92.2273 | 20.6212 |
| 6 | 132.967 | 92.6022 | 19.817 |
| 7 | 131.728 | 93.0686 | 19.8256 |
| 8 | 131.243 | 93.1954 | 21.5905 |
| 9 | 129.931 | 94.9256 | 22.5219 |
| 10 | 128.326 | 94.0692 | 22.372 |
| 11 | 127.388 | 93.1978 | 22.2945 |
| 12 | 127.302 | 92.9861 | 24.1133 |
| 13 | 126.715 | 92.5921 | 23.0543 |
| 14 | 125.769 | 92.2719 | 23.6588 |
| 15 | 125.34 | 92.1534 | 24.4865 |
| 16 | 124.51 | 91.5898 | 24.6737 |
| 17 | 123.909 | 91.3027 | 24.2519 |
| 18 | 123.14 | 90.8428 | 24.0024 |
| 19 | 122.838 | 90.5193 | 25.3023 |
| 20 | 122.236 | 90.1906 | 23.7715 |
| 21 | 121.391 | 89.1282 | 26.3195 |
| 22 | 120.445 | 88.9261 | 22.4497 |
| 23 | 119.939 | 88.0045 | 23.5392 |

Table S3.The control points of B-spline fitting ACor of NO.47 data

| ***ACo*** | | | |
| --- | --- | --- | --- |
| **NO.** | **x** | **y** | **z** |
| 1 | 118.143 | 88.7683 | 21.8837 |
| 2 | 118.698 | 88.3519 | 22.4297 |
| 3 | 119.352 | 88.7517 | 23.1523 |
| 4 | 119.939 | 88.0045 | 23.5392 |

Table S4.The control points of B-spline fitting BA of NO.47 data

| ***BA*** | | | |
| --- | --- | --- | --- |
| **NO.** | **x** | **y** | **z** |
| 1 | 116.964 | 103.246 | 16.7064 |
| 2 | 117.136 | 103.736 | 15.2715 |
| 3 | 116.813 | 104.275 | 13.5021 |
| 4 | 116.749 | 105.389 | 11.5324 |
| 5 | 116.805 | 105.958 | 10.5955 |
| 6 | 116.768 | 106.52 | 9.56625 |
| 7 | 116.898 | 107.184 | 8.7354 |
| 8 | 116.939 | 107.781 | 7.89516 |
| 9 | 116.976 | 108.319 | 7.07776 |
| 10 | 117.009 | 108.938 | 6.30142 |
| 11 | 117.057 | 109.734 | 5.54262 |
| 12 | 117.139 | 110.634 | 4.68604 |
| 13 | 117.208 | 111.461 | 4.09565 |
| 14 | 116.831 | 111.768 | 1.91591 |
| 15 | 117.346 | 112.656 | 2.64279 |
| 16 | 117.743 | 112.784 | 3.5624 |
| 17 | 118.351 | 114.613 | 4.76424 |
| 18 | 120.135 | 115.742 | 4.28419 |
| 19 | 119.506 | 115.642 | 2.40435 |

Table S5.The control points of B-spline fitting MCAl of NO.47 data

| ***MCAl*** | | | |
| --- | --- | --- | --- |
| **NO.** | **x** | **y** | **z** |
| 1 | 101.677 | 90.8831 | 21.5646 |
| 2 | 102.111 | 90.9705 | 21.4235 |
| 3 | 102.417 | 91.3226 | 20.6707 |
| 4 | 103.283 | 91.2625 | 19.6794 |
| 5 | 104.091 | 92.5207 | 19.0838 |
| 6 | 105.334 | 91.5598 | 18.4001 |
| 7 | 106.163 | 91.9586 | 17.8877 |
| 8 | 106.659 | 91.8666 | 17.6464 |

Table S6.The control points of B-spline fitting MCAr of NO.47 data

| ***MCAr*** | | | |
| --- | --- | --- | --- |
| **NO.** | **x** | **y** | **z** |
| 1 | 137.219 | 90.5544 | 19.8926 |
| 2 | 136.997 | 90.134 | 18.3687 |
| 3 | 134.963 | 89.1851 | 14.5922 |
| 4 | 131.445 | 94.4401 | 17.3231 |
| 5 | 127.248 | 97.7068 | 17.4539 |
| 6 | 127.165 | 95.4202 | 14.3457 |

Table S7.The control points of B-spline fitting PCAl of NO.47 data

| ***PCAl*** | | | |
| --- | --- | --- | --- |
| **NO.** | **x** | **y** | **z** |
| 1 | 110.579 | 101.977 | 24.5005 |
| 2 | 110.918 | 102.03 | 24.4123 |
| 3 | 111.513 | 102.1 | 24.1187 |
| 4 | 112.537 | 102.15 | 24.5118 |
| 5 | 113.413 | 102.657 | 23.6729 |
| 6 | 113.869 | 102.211 | 22.5434 |
| 7 | 114.62 | 102.004 | 21.5239 |
| 8 | 115.175 | 102.026 | 20.3943 |
| 9 | 115.472 | 102.337 | 19.2595 |
| 10 | 115.967 | 102.568 | 17.8423 |
| 11 | 116.982 | 102.97 | 17.3674 |
| 12 | 116.964 | 103.246 | 16.7064 |

Table S8.The control points of B-spline fitting PCAr of NO.47 data

| ***PCAr*** | | | |
| --- | --- | --- | --- |
| **NO.** | **x** | **y** | **z** |
| 1 | 116.964 | 103.246 | 16.7064 |
| 2 | 117.174 | 103.045 | 17.218 |
| 3 | 117.812 | 102.873 | 17.9045 |
| 4 | 118.613 | 102.446 | 18.8957 |
| 5 | 119.28 | 102.439 | 19.8507 |
| 6 | 120.018 | 102.212 | 20.7386 |
| 7 | 121.025 | 102.142 | 21.3342 |
| 8 | 121.842 | 102.317 | 21.8254 |
| 9 | 122.401 | 102.411 | 23.1876 |
| 10 | 123.5 | 102.685 | 21.9993 |
| 11 | 123.728 | 102.833 | 21.8912 |

Table S9.The control points of B-spline fitting PCol of NO.47 data

| ***PCol*** | | | |
| --- | --- | --- | --- |
| **NO.** | **x** | **y** | **z** |
| 1 | 106.659 | 91.8666 | 17.6464 |
| 2 | 107.087 | 92.0657 | 17.3129 |
| 3 | 106.46 | 92.8525 | 17.9069 |
| 4 | 107.41 | 93.9642 | 17.813 |
| 5 | 107.942 | 95.1253 | 17.0275 |
| 6 | 108.034 | 96.4627 | 18.3796 |
| 7 | 108.933 | 97.5292 | 17.2707 |
| 8 | 108.922 | 98.289 | 18.1283 |
| 9 | 108.943 | 99.0917 | 18.5647 |
| 10 | 109.591 | 100.048 | 19.367 |
| 11 | 110.278 | 100.053 | 20.8181 |
| 12 | 111.665 | 101.104 | 23.2993 |
| 13 | 108.993 | 103.504 | 23.1744 |
| 14 | 110.579 | 101.977 | 24.5005 |

Table S10.The control points of B-spline fitting PCor of NO.47 data

| ***PCor*** | | | |
| --- | --- | --- | --- |
| **NO.** | **x** | **y** | **z** |
| 1 | 127.165 | 95.4202 | 14.3457 |
| 2 | 127.573 | 96.5417 | 13.9392 |
| 3 | 125.64 | 97.7397 | 13.9582 |
| 4 | 124.848 | 99.8431 | 15.1288 |
| 5 | 124.214 | 100.571 | 16.8235 |
| 6 | 124.27 | 101.128 | 18.8089 |
| 7 | 124.376 | 101.606 | 20.1827 |
| 8 | 124.109 | 102.238 | 20.8576 |
