# Supplementary material for: Skeleton-based cerebrovascular quantitative analysis
Source: BMC Med Imaging. 2016 Dec 20;16:68. doi: 10.1186/s12880-016-0170-8 (PMC5168872; doi:10.1186/s12880-016-0170-8)
Supplement: Additional file 6 — Normal047-Characters.docx. This file contains four tables (Table S11–S14). Table S11 records the curvature values of twenty sampling points of each vessel on the CoW. Table S12 records the torsion values of twenty sampling points of each vessel on the CoW. Table S13 records the radius values of twenty sampling points of each vessel on the CoW. Table S14 records the angle values of eighteen sampling points of each vessel on the CoW. The data of these four tables correspond to the Fig. 7 in the paper. And the data of the Fig. 8 are contained in all these four tables. (DOCX 26.9 kb) [file 12880_2016_170_MOESM6_ESM.docx]

Table S11.The curvature values of every vessel on the CoW of NO.47 data

| ***Curvature*** | | | | | | | | | | |
| --- | --- | --- | --- | --- | --- | --- | --- | --- | --- | --- |
|  | **ACAl** | **ACAr** | **ACo** | **BA** | **MCAl** | **MCAr** | **PCAl** | **PCAr** | **PCol** | **PCor** |
| 1 | 0.370 | 0.398 | 0.695 | 0.696 | 1.154 | 0.203 | 5.526 | 0.418 | 1.034 | 0.550 |
| 2 | 0.613 | 0.865 | 0.644 | 0.328 | 0.988 | 0.144 | 0.836 | 0.282 | 0.212 | 0.583 |
| 3 | 0.221 | 0.368 | 0.567 | 0.241 | 0.343 | 0.140 | 0.203 | 0.119 | 0.579 | 1.276 |
| 4 | 0.341 | 0.234 | 0.476 | 0.068 | 0.237 | 0.181 | 0.508 | 0.082 | 0.260 | 0.377 |
| 5 | 0.194 | 0.216 | 0.377 | 0.106 | 0.326 | 0.291 | 1.224 | 0.071 | 0.620 | 0.769 |
| 6 | 0.102 | 1.243 | 0.278 | 0.005 | 0.616 | 0.520 | 0.420 | 0.243 | 2.143 | 0.275 |
| 7 | 0.108 | 0.352 | 0.182 | 0.075 | 0.833 | 0.644 | 0.828 | 0.129 | 0.655 | 1.234 |
| 8 | 0.254 | 0.338 | 0.091 | 0.026 | 0.386 | 0.336 | 0.247 | 0.090 | 0.227 | 0.401 |
| 9 | 0.388 | 0.826 | 0.045 | 0.043 | 2.457 | 0.135 | 0.254 | 0.157 | 1.369 | 0.147 |
| 10 | 0.246 | 0.224 | 0.119 | 0.097 | 0.881 | 0.036 | 0.156 | 0.233 | 0.313 | 0.143 |
| 11 | 0.271 | 1.304 | 0.213 | 0.093 | 0.752 | 0.028 | 0.203 | 0.268 | 0.454 | 0.192 |
| 12 | 0.937 | 0.654 | 0.312 | 0.040 | 3.651 | 0.072 | 0.229 | 0.201 | 0.529 | 0.255 |
| 13 | 0.681 | 0.770 | 0.412 | 0.472 | 0.334 | 0.091 | 0.260 | 0.212 | 0.186 | 0.148 |
| 14 | 1.007 | 0.929 | 0.511 | 0.033 | 1.123 | 0.136 | 0.073 | 0.532 | 0.120 | 0.052 |
| 15 | 5.167 | 0.191 | 0.601 | 1.220 | 0.552 | 0.235 | 0.105 | 0.172 | 0.306 | 0.070 |
| 16 | 1.529 | 0.065 | 0.673 | 0.499 | 0.421 | 0.444 | 0.283 | 2.517 | 0.838 | 0.207 |
| 17 | 0.669 | 4.208 | 0.715 | 0.369 | 0.187 | 0.720 | 0.514 | 0.680 | 0.290 | 0.253 |
| 18 | 0.153 | 3.493 | 0.721 | 0.510 | 0.088 | 0.634 | 0.143 | 0.184 | 0.514 | 0.323 |
| 19 | 0.048 | 0.214 | 0.690 | 0.971 | 0.313 | 0.314 | 0.690 | 1.006 | 2.917 | 0.243 |
| 20 | 0.250 | 0.661 | 0.628 | 0.265 | 0.532 | 0.136 | 0.827 | 0.949 | 0.113 | 0.855 |

Table S12.The torsion values of every vessel on the CoW of NO.47 data

| ***Torsion*** | | | | | | | | | | |
| --- | --- | --- | --- | --- | --- | --- | --- | --- | --- | --- |
|  | **ACAl** | **ACAr** | **ACo** | **BA** | **MCAl** | **MCAr** | **PCAl** | **PCAr** | **PCol** | **PCor** |
| 1 | 0.090 | 0.049 | -0.052 | 0.029 | 0.036 | 0.173 | 0.023 | -0.087 | 0.057 | 1.302 |
| 2 | 0.322 | 0.233 | -0.069 | 0.421 | 0.095 | 0.198 | 0.324 | -0.219 | 10.41 | 1.001 |
| 3 | 1.836 | 0.921 | -0.094 | -0.116 | 0.674 | 0.182 | -31.354 | -1.198 | -0.384 | -0.514 |
| 4 | -0.717 | 0.756 | -0.136 | 1.973 | 1.305 | 0.138 | -0.205 | -2.476 | -1.913 | -2.416 |
| 5 | 1.340 | 1.969 | -0.216 | 0.309 | 5.059 | 0.094 | 1.160 | -3.524 | 1.623 | -0.387 |
| 6 | 0.384 | 0.248 | -0.390 | 21.90 | 1.473 | 0.061 | -1.826 | -0.312 | 0.336 | -3.295 |
| 7 | -1.578 | 0.982 | -0.901 | 0.856 | 0.561 | -0.014 | -0.101 | -0.540 | 1.297 | -0.081 |
| 8 | -0.369 | 1.259 | -3.584 | -6.015 | 2.511 | -0.026 | -0.862 | -1.098 | 6.677 | -0.254 |
| 9 | -0.364 | 0.026 | -14.800 | -1.591 | 0.166 | -0.066 | -0.819 | 1.835 | -0.258 | -1.415 |
| 10 | -0.351 | 0.141 | -2.106 | 0.157 | 1.147 | -0.487 | -1.871 | 0.842 | -2.476 | 0.958 |
| 11 | 0.708 | 0.081 | -0.667 | 0.338 | 1.672 | -0.557 | -1.131 | 0.686 | -0.903 | 0.840 |
| 12 | -0.231 | -5.011 | -0.318 | -0.809 | -0.286 | -0.045 | 0.140 | 1.218 | -0.017 | 0.502 |
| 13 | -0.192 | -0.176 | -0.185 | -0.198 | -7.416 | -0.032 | -0.022 | 4.200 | -0.123 | 0.059 |
| 14 | -0.153 | -0.435 | -0.121 | -8.416 | -0.448 | -0.022 | -0.275 | 0.624 | -0.157 | 0.430 |
| 15 | -0.228 | 1.142 | -0.085 | 0.257 | -2.206 | -0.015 | -3.638 | 0.333 | 0.391 | 0.535 |
| 16 | -0.269 | -30.970 | -0.063 | -0.534 | -0.222 | -0.010 | -0.516 | 0.007 | 0.099 | 0.066 |
| 17 | -0.190 | 0.060 | -0.049 | -1.038 | -1.135 | -0.007 | -0.438 | 0.892 | 0.718 | 1.471 |
| 18 | -0.178 | 0.054 | -0.039 | 0.438 | -5.115 | -0.005 | -5.375 | -6.482 | 0.877 | 0.882 |
| 19 | -1.409 | 0.490 | -0.031 | 0.187 | -0.409 | -0.004 | -0.282 | -0.504 | 0.144 | -2.198 |
| 20 | -0.066 | 0.016 | -0.026 | 0.065 | -0.131 | -0.003 | -0.082 | -0.159 | 0.064 | -0.157 |

Table S13.The radius values of every vessel on the CoW of NO.47 data

| ***Radius(mm)*** | | | | | | | | | | |
| --- | --- | --- | --- | --- | --- | --- | --- | --- | --- | --- |
|  | **ACAl** | **ACAr** | **ACo** | **BA** | **MCAl** | **MCAr** | **PCAl** | **PCAr** | **PCol** | **PCor** |
| 1 | 2.657 | 2.923 | 2.141 | 4.421 | 2.657 | 2.923 | 2.361 | 4.421 | 3.048 | 2.986 |
| 2 | 3.292 | 2.501 | 1.918 | 4.286 | 2.778 | 2.659 | 2.044 | 4.081 | 3.033 | 2.844 |
| 3 | 2.435 | 2.422 | 1.782 | 3.598 | 3.041 | 2.196 | 1.790 | 3.800 | 2.254 | 2.403 |
| 4 | 1.389 | 2.569 | 1.724 | 2.974 | 3.280 | 1.639 | 1.820 | 3.459 | 1.456 | 1.829 |
| 5 | 1.256 | 2.996 | 1.732 | 3.007 | 3.334 | 1.087 | 1.839 | 2.957 | 0.942 | 1.286 |
| 6 | 1.386 | 3.794 | 1.797 | 3.055 | 3.176 | 0.643 | 1.831 | 2.429 | 0.965 | 0.939 |
| 7 | 1.047 | 3.883 | 1.907 | 2.899 | 2.980 | 0.407 | 1.902 | 2.150 | 1.249 | 0.935 |
| 8 | 0.823 | 2.721 | 2.053 | 2.972 | 2.937 | 0.417 | 2.020 | 2.146 | 1.256 | 1.200 |
| 9 | 0.983 | 1.452 | 2.224 | 2.899 | 3.156 | 0.605 | 2.109 | 2.214 | 1.234 | 1.530 |
| 10 | 1.194 | 1.333 | 2.409 | 2.750 | 3.498 | 0.895 | 2.159 | 2.222 | 1.264 | 1.718 |
| 11 | 1.236 | 2.364 | 2.598 | 2.646 | 3.774 | 1.211 | 2.209 | 2.186 | 1.213 | 1.647 |
| 12 | 1.218 | 2.523 | 2.781 | 2.843 | 3.799 | 1.476 | 2.246 | 2.140 | 1.277 | 1.428 |
| 13 | 1.257 | 2.186 | 2.947 | 3.417 | 3.501 | 1.645 | 2.202 | 2.145 | 1.229 | 1.205 |
| 14 | 1.158 | 2.112 | 3.085 | 3.111 | 3.053 | 1.747 | 2.120 | 2.247 | 1.319 | 1.092 |
| 15 | 1.102 | 2.201 | 3.186 | 2.661 | 2.659 | 1.818 | 2.180 | 2.348 | 1.820 | 1.107 |
| 16 | 1.494 | 2.253 | 3.239 | 2.804 | 2.505 | 1.894 | 2.482 | 2.306 | 2.426 | 1.247 |
| 17 | 1.125 | 2.360 | 3.233 | 1.652 | 2.592 | 2.012 | 2.971 | 2.200 | 2.642 | 1.477 |
| 18 | 1.313 | 2.331 | 3.158 | 1.242 | 2.795 | 2.209 | 3.554 | 2.181 | 2.497 | 1.729 |
| 19 | 1.516 | 2.196 | 3.004 | 1.634 | 2.989 | 2.521 | 4.088 | 2.278 | 2.300 | 1.936 |
| 20 | 2.141 | 2.759 | 2.759 | 1.613 | 3.048 | 2.986 | 4.421 | 2.509 | 2.361 | 2.029 |

Table S14.The angle values of every vessel on the CoW of NO.47 data

| ***Angle*** | | | | | | | | | | |
| --- | --- | --- | --- | --- | --- | --- | --- | --- | --- | --- |
|  | **ACAl** | **ACAr** | **ACo** | **BA** | **MCAl** | **MCAr** | **PCAl** | **PCAr** | **PCol** | **PCor** |
| 1 | 2.252 | 2.462 | 2.988 | 2.681 | 2.944 | 2.025 | 2.446 | 2.577 | 2.508 | 1.965 |
| 2 | 2.539 | 2.450 | 3.025 | 2.661 | 3.001 | 2.022 | 2.867 | 2.623 | 2.594 | 2.091 |
| 3 | 2.630 | 2.409 | 3.059 | 2.653 | 2.922 | 2.019 | 2.467 | 2.638 | 2.525 | 2.344 |
| 4 | 2.527 | 2.343 | 3.083 | 2.638 | 2.870 | 2.028 | 2.205 | 2.621 | 2.385 | 2.348 |
| 5 | 2.354 | 2.293 | 3.083 | 2.638 | 2.856 | 2.062 | 2.265 | 2.587 | 2.228 | 2.195 |
| 6 | 2.192 | 2.290 | 3.059 | 2.622 | 2.846 | 2.139 | 2.355 | 2.553 | 2.325 | 2.104 |
| 7 | 2.052 | 2.155 | 3.024 | 2.612 | 2.794 | 2.272 | 2.476 | 2.513 | 2.258 | 2.135 |
| 8 | 1.960 | 2.083 | 2.986 | 2.597 | 2.730 | 2.455 | 2.575 | 2.460 | 2.154 | 2.213 |
| 9 | 1.901 | 2.158 | 2.947 | 2.557 | 2.713 | 2.676 | 2.617 | 2.401 | 2.198 | 2.282 |
| 10 | 1.835 | 2.264 | 2.907 | 2.520 | 2.662 | 2.909 | 2.632 | 2.349 | 2.192 | 2.342 |
| 11 | 1.826 | 2.280 | 2.867 | 2.482 | 2.618 | 3.017 | 2.652 | 2.305 | 2.197 | 2.407 |
| 12 | 1.894 | 2.304 | 2.828 | 2.428 | 2.678 | 2.809 | 2.687 | 2.255 | 2.255 | 2.491 |
| 13 | 1.917 | 2.229 | 2.790 | 2.187 | 2.763 | 2.561 | 2.742 | 2.165 | 2.338 | 2.593 |
| 14 | 2.087 | 2.265 | 2.754 | 2.014 | 2.833 | 2.317 | 2.801 | 1.958 | 2.371 | 2.706 |
| 15 | 2.052 | 2.227 | 2.719 | 2.344 | 2.885 | 2.098 | 2.833 | 1.702 | 2.322 | 2.823 |
| 16 | 2.166 | 2.257 | 2.687 | 2.685 | 2.902 | 1.921 | 2.820 | 1.599 | 2.194 | 2.927 |
| 17 | 2.248 | 2.053 | 2.658 | 2.794 | 2.884 | 1.796 | 2.760 | 1.547 | 1.839 | 3.000 |
| 18 | 2.278 | 2.258 | 2.633 | 2.502 | 2.840 | 1.728 | 2.616 | 1.408 | 1.581 | 3.010 |
